# (*S*)-4-(2-Chloro­propan-2-yl)-1-(2,2,2-trichloro­eth­yl)cyclo­hexene

**DOI:** 10.1107/S1600536811010257

**Published:** 2011-03-23

**Authors:** Brahim Boualy, Mohamed Anoir Harrad, Larbi El Firdoussi, Mustapha Ait Ali, Corrado Rizzoli

**Affiliations:** a Equipe de Chimie de Coordination et Catalyse, Faculté des Sciences-Semlalia, BP 2390, 40001 Marrakech, Morocco; b Dipartimento di Chimica Generale ed Inorganica, Chimica Analitica, Chimica Fisica, Universitá degli Studi di Parma, Viale G. P. Usberti 17/A, I-43124 Parma, Italy

## Abstract

The title compound, C_11_H_16_Cl_4_, was synthesized by the reaction of (1*S*)-β-pinene with triethyl­amine in the presence of ZnCl_2_. The cyclo­hexene ring assumes a half-boat conformation. The crystal packing is governed only by van der Waals inter­actions. The structure, which has been refined in *P*2_1_, presents a striking *P*2_1_/*m* pseudosymmetry.

## Related literature

For background to the synthesis of polyhalogenated compounds, see: Delaude *et al.* (2004 ▶); Borguet *et al.* (2007 ▶). For the synthesis and structure of natural chlorinated compounds reported by our group, see: Ziyat *et al.* (2002 ▶, 2004 ▶); Boualy *et al.* (2009 ▶). For bond-length data, see: Allen *et al.* (1987 ▶). For puckering parameters, see: Cremer & Pople (1975 ▶).
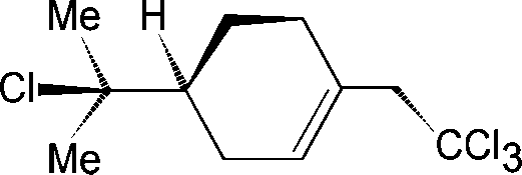


## Experimental

### 

#### Crystal data

C_11_H_16_Cl_4_
*M* *_r_* = 290.04
Monoclinic, 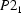

*a* = 10.6558 (7) Å
*b* = 10.3017 (6) Å
*c* = 6.3119 (3) Å
β = 91.251 (5)°
*V* = 692.71 (7) Å^3^
*Z* = 2
Cu *K*α radiation
μ = 7.50 mm^−1^
*T* = 294 K
0.21 × 0.09 × 0.07 mm

#### Data collection

Siemens AED diffractometer
Absorption correction: refined from Δ*F* (*DIFABS*; Walker & Stuart, 1983 ▶) *T*_min_ = 0.456, *T*_max_ = 0.601
2764 measured reflections
2528 independent reflections
2206 reflections with *I* > 2σ(*I*)
*R*_int_ = 0.038
3 standard reflections every 100 reflections  intensity decay: 0.02%

#### Refinement

*R*[*F* ^2^ > 2σ(*F* ^2^)] = 0.040
*wR*(*F* ^2^) = 0.129
*S* = 1.16
2528 reflections
136 parameters
1 restraint
H-atom parameters constrained
Δρ_max_ = 0.27 e Å^−3^
Δρ_min_ = −0.22 e Å^−3^
Absolute structure: Flack (1983 ▶); 1188 Friedel pairs
Flack parameter: −0.04 (3)

### 

Data collection: *AED* (Belletti *et al.*, 1993 ▶); cell refinement: *AED*; data reduction: *AED*; program(s) used to solve structure: *SIR97* (Altomare *et al.*, 1999 ▶); program(s) used to refine structure: *SHELXL97* (Sheldrick, 2008 ▶); molecular graphics: *ORTEP-3 for Windows* (Farrugia, 1997 ▶) and *SCHAKAL97* (Keller, 1997 ▶); software used to prepare material for publication: *SHELXL97* and *PARST95* (Nardelli, 1995 ▶).

## Supplementary Material

Crystal structure: contains datablocks global, I. DOI: 10.1107/S1600536811010257/bg2395sup1.cif

Structure factors: contains datablocks I. DOI: 10.1107/S1600536811010257/bg2395Isup2.hkl

Additional supplementary materials:  crystallographic information; 3D view; checkCIF report

## Figures and Tables

**Fig. 1.**
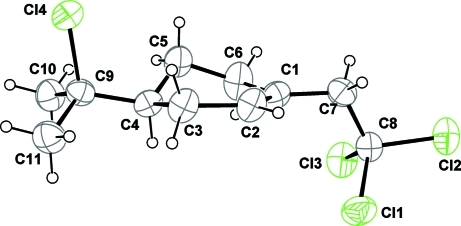
The molecular structure of the title compound, with displacement ellipsoids drawn at the 30% probability level.

**Fig. 2.**
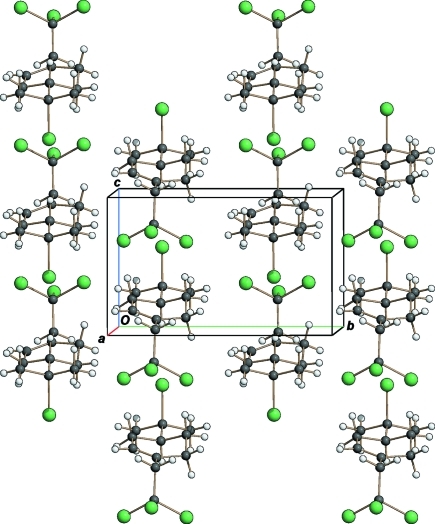
Crystal packing of the title compound approximately viewed along the a axis.

## supplementary crystallographic information

### Comment

The research on polyhalogenated alkanes, lactams and lactones, which are versatile intermediates in the synthesis of natural products and bioactive molecules, has held the attention of chemists for many years (Delaude *et al.*, 2004). Among others, the Kharasch reaction is an effective method for the formation of these polyhalogenated products. This process consists in the addition of a polyhalogenated alkane to an alkene and requires either a radical initiator or a transition metal catalyst (Borguet *et al.*, 2007). In the course of our ongoing research program aimed at the synthesis of natural chlorinated compounds (Ziyat *et al.*, 2002; Ziyat *et al.*, 2004; Boualy *et al.*, 2009), the title compound has been obtained and its crystal structure is reported herein.

In the molecule of the title compound (Fig. 1) all bond lengths (Allen *et al.*, 1987) and angles are normal. The cyclohexene ring assumes a half-boat conformation, with puckering parameters *Q*, θ and φ of 0.493 (4) Å, 51.9 (4)° and -142.0 (6)°, respectively (Cremer & Pople, 1975). The crystal structure (Fig. 2) is stabilized only by van der Waals interactions. The shortest intermolecular Cl···Cl separation observed is 3.5306 (11) Å (Cl2···Cl4^i^; symmetry code: (i) 1 + *x*, *y*, -1 + *z*). The structure, which has been  refined in P2_1_, presents a striking P2_1_/m pseudosymmetry (See refinement section for details).

### Experimental

A mixture of (1*S*)-β-pinene (1 g, 7.34 mmol) and triethylamine (1 ml, 7.11 mmol) in carbon tetrachloride (15 ml) was added to a solution of ZnCl_2_ in (1.1 g, 8.09 mmol) in water (15 ml) under stirring at room temperature. On completion of the reaction, the mixture was diluted with 25 ml of water, extracted with carbon tetrachloride (3 × 10 ml) and dried over Na_2_SO_4_. The title compound was isolated as a white powder by column chromatography on silica gel using *n*-hexane as eluent (yield 90%; m. p. = 48 °C), but colourless single crystals suitable for X-ray analysis were obtained by slow evaporation of a *n*-hexane solution. ^1^H NMR (300 MHz, CDCl_3_): δ p.p.m. 5.71 (m, 1H), 3.28 (s, 2H), 2.29 (m, 3H), 1.97 (m, 2H), 1.65 (m, 1H), 1.53 (s, 3H), 1.54 (s, 3H). ^13^C NMR (75 MHz, CDCl_3_): δ p.p.m. 131.03 (C_q_), 130.53 (CH═C), 99.06 (CCl_3_), 73.09 (CCl), 62.02 (CH_2_–CCl_3_), 45.73 (CH), 30.61 (CH_2_), 29.83 (CH_3_), 29.76 (CH_3_), 28.19 (CH_2_), 24.56 (CH_2_).

### Refinement

The molecule contains one chiral carbon atom at C4. Irrespective of this it is possible to solve the structure in the higher symmetry P2_1_/m space group, but this forces the molecule to have a crystallographically imposed mirror symmetry passing through C1 and C4 of the cyclohexene ring, resulting in the C2 (sp^2^) and C6 (sp^3^) carbon atoms to be symmetry-related and disordered over two orientations. This disorder is totally absent in the noncentrosymmetric P2_1_ space group. Moreover, refining in P2_1_/m results in significantly worse R values (R1 = 5.4%, wR2 = 18.2%). All H atoms were calculated geometrically and refined using a riding model, with C—H = 0.93–0.98 Å and with *U*_iso_(H) = 1.2 *U*_eq_(C) or 1.5 *U*_eq_(C) for methyl H atoms.

### Crystal data

**Table:**

C_11_H_16_Cl_4_	*F*(000) = 300
*M**_r_* = 290.04	*D*_x_ = 1.391 Mg m^−^^3^
Monoclinic, *P*2_1_	Cu *K*α radiation, λ = 1.54178 Å
Hall symbol: P 2yb	Cell parameters from 48 reflections
*a* = 10.6558 (7) Å	θ = 19.3–31.4°
*b* = 10.3017 (6) Å	µ = 7.50 mm^−^^1^
*c* = 6.3119 (3) Å	*T* = 294 K
β = 91.251 (5)°	Irregular block, colourless
*V* = 692.71 (7) Å^3^	0.21 × 0.09 × 0.07 mm
*Z* = 2	

### Data collection

**Table:**

Siemens AED diffractometer	2206 reflections with *I* > 2σ(*I*)
Radiation source: fine-focus sealed tube	*R*_int_ = 0.038
graphite	θ_max_ = 68.0°, θ_min_ = 4.2°
θ/2θ scans	*h* = −12→12
Absorption correction: part of the refinement model (Δ*F*) (*DIFABS*; Walker & Stuart, 1983)	*k* = −12→12
*T*_min_ = 0.456, *T*_max_ = 0.601	*l* = −1→7
2764 measured reflections	3 standard reflections every 100 reflections
2528 independent reflections	intensity decay: 0.02%

### Refinement

**Table:**

Refinement on *F*^2^	Secondary atom site location: difference Fourier map
Least-squares matrix: full	Hydrogen site location: inferred from neighbouring sites
*R*[*F*^2^ > 2σ(*F*^2^)] = 0.040	H-atom parameters constrained
*wR*(*F*^2^) = 0.129	*w* = 1/[σ^2^(*F*_o_^2^) + (0.0811*P*)^2^] where *P* = (*F*_o_^2^ + 2*F*_c_^2^)/3
*S* = 1.16	(Δ/σ)_max_ < 0.001
2528 reflections	Δρ_max_ = 0.27 e Å^−^^3^
136 parameters	Δρ_min_ = −0.22 e Å^−^^3^
1 restraint	Absolute structure: Flack (1983); 1188 Friedel pairs
Primary atom site location: structure-invariant direct methods	Flack parameter: −0.04 (3)

### Special details

**Table:**

Refinement. Refinement of *F*^2^ against ALL reflections. The weighted *R*-factor *wR* and goodness of fit *S* are based on *F*^2^, conventional *R*-factors *R* are based on *F*, with *F* set to zero for negative *F*^2^. The threshold expression of *F*^2^ > σ(*F*^2^) is used only for calculating *R*-factors(gt) *etc*. and is not relevant to the choice of reflections for refinement. *R*-factors based on *F*^2^ are statistically about twice as large as those based on *F*, and *R*- factors based on ALL data will be even larger.

### Fractional atomic coordinates and isotropic or equivalent isotropic displacement parameters (Å^2^)

**Table:**

	*x*	*y*	*z*	*U*_iso_*/*U*_eq_	
Cl1	0.92140 (14)	0.06856 (10)	−0.3180 (2)	0.0976 (4)	
Cl2	1.15231 (7)	0.20291 (18)	−0.23878 (15)	0.0990 (3)	
Cl3	0.92735 (12)	0.34740 (9)	−0.3247 (2)	0.0954 (4)	
Cl4	0.46844 (7)	0.21653 (15)	0.60646 (11)	0.0855 (3)	
C1	0.8287 (2)	0.2091 (4)	0.1030 (4)	0.0667 (6)	
C2	0.7742 (4)	0.1006 (3)	0.1658 (7)	0.0733 (9)	
H2	0.8213	0.0247	0.1630	0.088*	
C3	0.6416 (4)	0.0915 (3)	0.2416 (7)	0.0762 (11)	
H3A	0.6436	0.0713	0.3917	0.091*	
H3B	0.5996	0.0205	0.1683	0.091*	
C4	0.5658 (2)	0.2149 (4)	0.2064 (4)	0.0630 (5)	
H4	0.5460	0.2189	0.0542	0.076*	
C5	0.6483 (4)	0.3326 (4)	0.2565 (7)	0.0766 (11)	
H5A	0.5994	0.4114	0.2382	0.092*	
H5B	0.6776	0.3285	0.4029	0.092*	
C6	0.7592 (5)	0.3362 (4)	0.1129 (8)	0.0912 (13)	
H6A	0.8167	0.4032	0.1621	0.109*	
H6B	0.7302	0.3596	−0.0288	0.109*	
C7	0.9642 (2)	0.2108 (5)	0.0397 (4)	0.0721 (6)	
H7A	1.0063	0.1370	0.1052	0.086*	
H7B	1.0031	0.2887	0.0971	0.086*	
C8	0.9870 (2)	0.2066 (5)	−0.1959 (4)	0.0696 (6)	
C9	0.4387 (2)	0.2159 (4)	0.3195 (4)	0.0657 (6)	
C10	0.3606 (4)	0.3349 (4)	0.2697 (7)	0.0826 (11)	
H10A	0.4088	0.4115	0.3014	0.124*	
H10B	0.2866	0.3341	0.3539	0.124*	
H10C	0.3367	0.3347	0.1222	0.124*	
C11	0.3614 (5)	0.0931 (4)	0.2681 (8)	0.0870 (13)	
H11A	0.4109	0.0175	0.3001	0.131*	
H11B	0.3379	0.0929	0.1204	0.131*	
H11C	0.2872	0.0924	0.3518	0.131*	

### Atomic displacement parameters (Å^2^)

**Table:**

	*U*^11^	*U*^22^	*U*^33^	*U*^12^	*U*^13^	*U*^23^
Cl1	0.1090 (9)	0.0869 (6)	0.0972 (9)	−0.0153 (6)	0.0089 (8)	−0.0282 (6)
Cl2	0.0705 (4)	0.1114 (7)	0.1159 (6)	0.0073 (6)	0.0184 (4)	0.0098 (8)
Cl3	0.0994 (8)	0.0875 (7)	0.0998 (8)	0.0092 (6)	0.0124 (7)	0.0294 (6)
Cl4	0.0896 (5)	0.1015 (6)	0.0658 (4)	−0.0017 (7)	0.0100 (3)	−0.0054 (6)
C1	0.0785 (14)	0.0560 (12)	0.0656 (13)	0.011 (2)	0.0025 (11)	−0.002 (2)
C2	0.084 (2)	0.0530 (17)	0.083 (2)	0.0111 (15)	0.0145 (18)	0.0110 (15)
C3	0.090 (3)	0.0472 (18)	0.092 (3)	0.0030 (16)	0.020 (2)	0.0037 (17)
C4	0.0766 (14)	0.0513 (12)	0.0611 (12)	0.0043 (18)	0.0036 (10)	0.0010 (18)
C5	0.079 (2)	0.0523 (17)	0.099 (3)	−0.0007 (16)	0.013 (2)	−0.009 (2)
C6	0.091 (2)	0.0473 (15)	0.136 (4)	0.0015 (16)	0.028 (3)	0.008 (2)
C7	0.0728 (14)	0.0695 (14)	0.0736 (14)	0.000 (2)	−0.0061 (11)	0.007 (2)
C8	0.0673 (13)	0.0629 (13)	0.0787 (15)	0.0014 (19)	0.0050 (11)	0.005 (2)
C9	0.0712 (14)	0.0567 (13)	0.0691 (13)	−0.0005 (18)	−0.0036 (10)	−0.0042 (19)
C10	0.078 (3)	0.071 (2)	0.099 (3)	0.0121 (19)	0.002 (2)	0.001 (2)
C11	0.089 (3)	0.071 (3)	0.100 (4)	−0.007 (2)	−0.001 (3)	−0.008 (2)

### Geometric parameters (Å, °)

**Table:**

Cl1—C8	1.755 (4)	C5—H5A	0.9700
Cl2—C8	1.789 (3)	C5—H5B	0.9700
Cl3—C8	1.774 (4)	C6—H6A	0.9700
Cl4—C9	1.832 (3)	C6—H6B	0.9700
C1—C2	1.324 (5)	C7—C8	1.512 (4)
C1—C6	1.506 (5)	C7—H7A	0.9700
C1—C7	1.507 (4)	C7—H7B	0.9700
C2—C3	1.504 (6)	C9—C10	1.511 (5)
C2—H2	0.9300	C9—C11	1.541 (6)
C3—C4	1.520 (5)	C10—H10A	0.9600
C3—H3A	0.9700	C10—H10B	0.9600
C3—H3B	0.9700	C10—H10C	0.9600
C4—C5	1.526 (5)	C11—H11A	0.9600
C4—C9	1.545 (4)	C11—H11B	0.9600
C4—H4	0.9800	C11—H11C	0.9600
C5—C6	1.505 (6)		
			
C2—C1—C6	120.1 (3)	C1—C7—C8	115.8 (2)
C2—C1—C7	121.2 (4)	C1—C7—H7A	108.3
C6—C1—C7	118.5 (4)	C8—C7—H7A	108.3
C1—C2—C3	124.7 (3)	C1—C7—H7B	108.3
C1—C2—H2	117.7	C8—C7—H7B	108.3
C3—C2—H2	117.7	H7A—C7—H7B	107.4
C2—C3—C4	113.6 (3)	C7—C8—Cl1	112.6 (3)
C2—C3—H3A	108.8	C7—C8—Cl3	111.3 (3)
C4—C3—H3A	108.8	Cl1—C8—Cl3	109.02 (15)
C2—C3—H3B	108.8	C7—C8—Cl2	109.21 (18)
C4—C3—H3B	108.8	Cl1—C8—Cl2	107.5 (2)
H3A—C3—H3B	107.7	Cl3—C8—Cl2	107.0 (2)
C3—C4—C5	109.4 (2)	C10—C9—C11	109.4 (2)
C3—C4—C9	114.0 (3)	C10—C9—C4	113.2 (3)
C5—C4—C9	113.9 (3)	C11—C9—C4	111.5 (3)
C3—C4—H4	106.3	C10—C9—Cl4	106.6 (2)
C5—C4—H4	106.3	C11—C9—Cl4	106.9 (3)
C9—C4—H4	106.3	C4—C9—Cl4	108.81 (17)
C6—C5—C4	110.5 (3)	C9—C10—H10A	109.5
C6—C5—H5A	109.5	C9—C10—H10B	109.5
C4—C5—H5A	109.5	H10A—C10—H10B	109.5
C6—C5—H5B	109.5	C9—C10—H10C	109.5
C4—C5—H5B	109.5	H10A—C10—H10C	109.5
H5A—C5—H5B	108.1	H10B—C10—H10C	109.5
C5—C6—C1	113.4 (3)	C9—C11—H11A	109.5
C5—C6—H6A	108.9	C9—C11—H11B	109.5
C1—C6—H6A	108.9	H11A—C11—H11B	109.5
C5—C6—H6B	108.9	C9—C11—H11C	109.5
C1—C6—H6B	108.9	H11A—C11—H11C	109.5
H6A—C6—H6B	107.7	H11B—C11—H11C	109.5
